# Heritability—a paradox of quantitative genetics

**DOI:** 10.1093/genetics/iyag232

**Published:** 2026-08-31

**Authors:** Shizhong Xu

**Affiliations:** Department of Botany and Plant Sciences, University of California Riverside, Riverside, CA 92521, United States

**Keywords:** base population, current population, fixed model, heritability, pedigree density coefficient, pedigree sparsity coefficient, random model

## Abstract

Heritability is the foundation of quantitative genetics, yet the way it is defined often conflicts with how it is estimated. On one hand, heritability represents the proportion of phenotypic variance explained by genetic variance in the population from which data are sampled. On the other hand, heritability estimated with family and pedigree data represents the heritability in a hypothetical base population where all individuals are assumed to be independent and noninbred. The discrepancy may not be obvious to many people in the quantitative genetics community. This study demonstrates the discrepancy and, more importantly, introduces a pedigree sparsity coefficient (PSC) to correct the genetic variance/heritability from the base population to the current population. For very dense pedigree data, the correction may allow breeders to better understand the genetic basis of the trait of the current population and predict genetic gain for selection within the current population. The theory and method have been validated with simulated data and data collected from a long-term selection experiment in house mice. The PSC also applies to genomic heritability, where the pedigree relationship is replaced by a genomic relationship matrix calculated from genome-wide markers.

## Introduction

Heritability estimate represents somewhat of a genetic paradox. Sampled family or pedigree data must be used to estimate the genetic variance (Fisher 1918). However, the estimated genetic variance does not represent the variance of the current population (Careau et al. 2013). A simple example will suffice to demonstrate the paradox. Suppose that we sample *m* monozygotic twins of unrelated parents from a reference or base population. The sample size is n=2m, but we only have *m* independent genetic values in the population. The genetic variance should be around


(1) $$\sigma_{A}^{2}=\frac{1}{m-1}\overset{m}{\underset{\;j=1}{\sum }}\;(a_{j}-\bar{a}{)}^{2}$$

where $a_{j}$ is the genetic value of the twins in the *j*th family and $\bar{a}$ is the mean of the genetic value of the population. However, the actual genetic variance of the current population, when we consider twins as 2 individuals, is


(2) $$\sigma_{MZT}^{2}=\;\frac{1}{2m-1}\overset{m}{\underset{\;j=1}{\sum }}\;2(a_{j}-\bar{a}{)}^{2}=\;\frac{2}{2m-1}\times \frac{m-1}{m-1}\overset{m}{\underset{\;j=1}{\sum }}\;(a_{j}-\bar{a}{)}^{2}=\;\frac{2(m-1)}{2m-1}\sigma_{A}^{2}$$

For example, if m=10, then $\sigma_{MZT}^{2}=0.9474\sigma_{A}^{2}$. This reduction in genetic variance appears to be trivial in twins but may not be ignored in other types of population. A general formula for the reduction in genetic variance depends on the pedigree density coefficient (PDC) or pedigree sparsity coefficient (PSC), where PSC=1−PDC for 0≤PSC≤1. A dense pedigree is defined as a pedigree whose individuals are connected closely while a sparse pedigree is one where individuals are remotely related. The PSC allows us to investigate the relationship between the genetic variance of the current population and a hypothetical “base” population.

In classical quantitative genetics, the genetic variance of multiple independent loci under Hardy-Weinberg equilibrium is defined as


(3) $$\sigma_{A}^{2}=\overset{m}{\underset{k=1}{\sum }}\;2p_{k}(1-p_{k})\alpha_{k}^{2}$$

where $p_{k}$ is the frequency of the reference allele of locus *k* and $\alpha_{k}$ is the average effect of gene substitution of that locus (Falconer and Mackay 1996; Lynch and Walsh 1998; Walsh et al. 2025). This genetic variance is based on a fixed model framework where the average effects of gene substitution are treated as fixed effects and genotype indicator array of loci are treated as random variables (de los Campos et al. 2015). Heritability of a trait based on this genetic variance represents the heritability in this population. However, the genetic variance of the current population cannot be estimated in classical quantitative genetics until genomic data are available because the genetic relationship matrix can be replaced with a marker-inferred relationship matrix.

Family and pedigree data are commonly used to estimate trait heritability (Falconer and Mackay 1996; Lynch and Walsh 1998). The estimated heritability is supposed to facilitate prediction of genetic gain after selection, ie the breeders’ equation (Lush 1937, 1940, 1945), but such prediction is biased upward. Two approaches may be considered to estimate the heritability in the current population. One is to take the difference between the phenotypic variance and the environmental variance because the estimated environmental variance is unbiased. This approach does not have a predictive value because it depends on the estimated environmental variance. We do not know, a priori, how much reduction in genetic variance we can expect. The second approach is to adjust the genetic variance of the base population to the genetic variance of the current population with PSC.

Some studies showed that heritability estimated from pedigree studies in animals agree well with heritability estimated from response to artificial selection (realized heritability), suggesting that estimates from family studies are not necessarily inflated (Manolio et al. 2009). However, such biases are not easily recognized from one or a few selection experiments. It requires a meta-analysis summarizing a large number of experimental results. Even a widely cited review that clarifies the definition of heritability, its applications, and common misunderstandings, specifically bridging the gap between traditional quantitative genetics and modern genomics did not recognize the reduction in heritability from the base population to the current population (Visscher et al. 2008).

Genomic heritability is a new concept driven by genomic data (VanRaden 2008; Yang et al. 2010; de los Campos et al. 2015). The genetic relationship (identical by descent, IBD) matrix in classical quantitative genetics is replaced by a marker inferred relationship (identical by state, IBS) matrix. One advantage of using this IBS matrix is that heritability can be estimated without family or pedigree data. This is particularly important for beef cattle and wild animals where pedigree data are often not available. Genome-wide marker data are used in 2 different ways. One way is through genome-wide association studies where statistically significant markers are used to fit a multiple regression model to the trait of interest and genomic heritability is estimated by the *R*-squared or adjusted *R*-squared. Since only significant markers are included, this genomic heritability often account a proportion of the narrow-sense heritability, a phenomenon called missing heritability (Manolio et al. 2009; Yang et al. 2010, 2015). Another way is through genomic selection where all markers are included in the model as random effects (Meuwissen et al. 2001; VanRaden 2008). Markers enter the model in the form of IBS matrix. Data are analyzed via the typical linear mixed model (Henderson 1953, 1975), delivering an estimated polygenic variance and a residual variance (Patterson and Thompson 1971). Genomic heritability is then defined as the ratio of the polygenic variance to the sum of the polygenic variance and the residual variance. This genomic heritability does not represent the heritability of the current population, although the IBS matrix is inferred from markers of the current population. An obvious support of this argument is that the polygenic variance and the residual variance do not add up to the phenotypic variance of the current population. The proposed PSC for adjusting the genetic variance from the base population to the current population applies to genomic heritability equally well.

This study has 2 specific aims: (i) recognizing the discrepancy between the estimated heritability from family/pedigree data and the heritability of the current population from which the data are sampled; (ii) deriving a pedigree sparsity coefficient to quantify the discrepancy under different types of populations.

## Theory and methods

### Genetic variance of a base population

Let *y* be an n×1 vector of phenotypic values from a sample. The mixed model for pedigree data is


(4) y=Xβ+a+ε

where *X* is a design matrix for fixed effects, *β* is a vector of the fixed effects, $a∼N(0,A\sigma_{A}^{2})$ is an n×1 vector of additive genetic effects (polygenic effects) and $\varepsilon ∼N(0,I\sigma_{E}^{2})$ is a vector of environmental effects. This is an additive model assuming absence of dominance and epistatic effects. The expectation of the model is E(y)=Xβ and the variance is


(5) $$\mathrm{var}(y)=V=\mathrm{var}(a)+\mathrm{var}(\varepsilon )=A\sigma_{A}^{2}+I\sigma_{E}^{2}$$

where *A* is the additive genetic relationship matrix (2 times the coancestry matrix), $\sigma_{A}^{2}$ is the additive genetic variance and $\sigma_{E}^{2}$ is the environmental variance. In animal breeding, *A* is also called the numerator relationship matrix. The conventional heritability is defined as


(6) $$h^{2}=\frac{\sigma_{A}^{2}}{\sigma_{A}^{2}+\sigma_{E}^{2}}$$

The polygenic variance represents the genetic variance of a hypothetical “base population” in which all individuals are genetically independent and noninbred. The base population is also called the founder population, which is defined arbitrarily by the investigator. It is logically confusing with this definition of heritability when we estimate the environmental variance from the current population but combine the estimated genetic variance from a hypothetical base population to build the heritability. The model for family data is a special case of the pedigree model where the numerator relationship matrix has a systematic pattern. For example, if a population contains 2 sire families, each sire is mated to 2 dams and each dam produces 2 offspring, then the numerator relationship matrix is


(7) $$A=\left[\begin{array}{cccccccc} 1 & 0.5 & 0.25 & 0.25 & 0 & 0 & 0 & 0\\ 0.5 & 1 & 0.25 & 0.25 & 0 & 0 & 0 & 0\\ 0.25 & 0.25 & 1 & 0.5 & 0 & 0 & 0 & 0\\ 0.25 & 0.25 & 0.5 & 1 & 0 & 0 & 0 & 0\\ 0 & 0 & 0 & 0 & 1 & 0.5 & 0.25 & 0.25\\ 0 & 0 & 0 & 0 & 0.5 & 1 & 0.25 & 0.25\\ 0 & 0 & 0 & 0 & 0.25 & 0.25 & 1 & 0.5\\ 0 & 0 & 0 & 0 & 0.25 & 0.25 & 0.5 & 1 \end{array}\right]$$

With the above family data, the estimated genetic variance represents the genetic variance of the parents, not the genetic variance of the progeny (the current population).

### Genetic variance of the current population

#### General formula

Let us perform eigen decomposition for the numerator relationship matrix, $A=UDU^{T}$, where *D* is a diagonal matrix for the eigenvalues and *U* is an orthogonal matrix of eigenvectors. Let $Z=UD^{\frac{1}{2}}$ and rewrite the model in the following form,


(8) y=Xβ+Zγ+ε

where $\gamma ∼N(0,I\sigma_{A}^{2})$ is a vector of normal variables. Models (4) and (8) are equivalent because they have the same expectation and the same variance, as demonstrated below,


(9) $$\mathrm{var}(y)=V=ZZ^{T}\sigma_{A}^{2}+I\sigma_{E}^{2}=UD^{\frac{1}{2}}D^{\frac{1}{2}}U^{T}\sigma_{A}^{2}+I\sigma_{E}^{2}=A\sigma_{A}^{2}+I\sigma_{E}^{2}$$

This is a mixed model because we have treated *γ* as random effects. If we want to evaluate the variance of the current population, *γ* must be treated as fixed effects, at least in the initial stage of the analysis. In the meantime, *Z* are considered as variables to reflect the variation among members within the current population. To derive the genetic variance of the current population, we must first remove the fixed effects with an annihilator matrix,


(10) $$M=I-X(X^{T}X{)}^{-1}X^{T}$$

The fixed effects are removed if we multiply both sides of equation (8) by *M*, leading to


(11) My=MZγ+Mε

Let us construct various quadratic forms,


(12) $$y^{T}My=\gamma^{T}Z^{T}MZ\gamma +\varepsilon^{T}M\varepsilon$$

where $y^{T}My=SS_{T}$ is the total sum of squares, $\gamma^{T}Z^{T}MZ\gamma =SS_{\gamma }$ is the genetic sum of squares, and $\varepsilon^{T}M\varepsilon =SS_{\varepsilon }$ is the environmental sum of squares. Let us take the expectations of the quadratic forms with respect to $\gamma ∼N(0,I\sigma_{A}^{2})$ and $\varepsilon ∼N(0,I\sigma_{E}^{2})$, yielding


(13) $$E(y^{T}My)=E(\gamma^{T}Z^{T}MZ\gamma )+E(\varepsilon^{T}M\varepsilon )$$

where


(14) $$E(\gamma^{T}Z^{T}MZ\gamma )=\mathrm{tr}[\mathrm{var}(\gamma )Z^{T}MZ]=\mathrm{tr}(Z^{T}MZ)\sigma_{A}^{2}=\mathrm{tr}(ZZ^{T}M)\sigma_{A}^{2}=\mathrm{tr}(AM)\sigma_{A}^{2}$$

and


(15) $$E(\varepsilon^{T}M\varepsilon )=\mathrm{tr}[\mathrm{var}(\varepsilon )M]=\mathrm{tr}(M)\sigma_{E}^{2}$$

Note that $ZZ^{T}=A$ is the numerator relationship matrix, as shown in equation (9). The total phenotypic sum of squares divided by the degree of freedom, tr(M), becomes the phenotypic variance of the current population.


(16) $$\sigma_{P}^{2}=\;\frac{y^{T}My}{\mathrm{tr}(M)}\;=\frac{1}{\mathrm{tr}(M)}[\mathrm{tr}(AM)\sigma_{A}^{2}+\mathrm{tr}(M)\sigma_{E}^{2}]=\rho \sigma_{A}^{2}+\sigma_{E}^{2}$$

where


(17) $$\rho =\;\frac{\mathrm{tr}(AM)}{\mathrm{tr}(M)}$$

is the pedigree sparsity coefficient and 1−ρ is the pedigree density coefficient. If all individuals in the current population are independent and noninbred, *A* would be an identity matrix and *ρ* would be 1, representing the maximum sparsity and 1−ρ represents the maximum density. However, if A=I, the genetic variance and environmental variance are confounded, and we would not be able to estimate the genetic variance.

The heritability of the trait in the current population is defined as


(18) $$h_{C}^{2}=\;\frac{\rho \sigma_{A}^{2}}{\rho \sigma_{A}^{2}+\sigma_{E}^{2}}$$

which can be reformulated as


(19) $$h_{C}^{2}=\frac{\rho \sigma_{A}^{2}/(\sigma_{A}^{2}+\sigma_{E}^{2})}{\rho \sigma_{A}^{2}/(\sigma_{A}^{2}+\sigma_{E}^{2})+\sigma_{E}^{2}/(\sigma_{A}^{2}+\sigma_{E}^{2})}=\frac{\rho h^{2}}{1+\rho h^{2}-h^{2}}=\;\frac{\rho }{1-(1-\rho )h^{2}}h^{2}$$

The above derivation is from the ANOVA point of view by partitioning the total variance into a genetic variance and a residual variance. A reviewer suggested directly taking the expectation of the quadratic form of *y*


(20) $$E(y^{T}My)=\mathrm{tr}(MV)=\mathrm{tr}[M(ZZ^{T}\sigma_{A}^{2}+I\sigma_{E}^{2})]=\mathrm{tr}(AM)\sigma_{A}^{2}+\mathrm{tr}(M)\sigma_{E}^{2}$$

which is also the sum of equations (14) and (15). This derivation is based on the mixed model theory. The derivation allows us to connect $\mathrm{tr}(AM)\sigma_{A}^{2}$ with the genetic sum of squares, and thus, gives an alternative derivation of equation (14).

#### A special case

In a special case where the fixed effect contains only the intercept, X=1, a unity vector of length *n*, the *M* matrix is


(21) $$M=I-X(X^{T}X{)}^{-1}X^{T}=I-\frac{1}{n}11^{\text{T}}$$

where *n* is the population size. This special case leads to tr(M)=n−1 and


(22) $$\mathrm{tr}(AM)=\mathrm{tr}(A)-\frac{1}{n}1^{T}A1=\overset{n}{\underset{i=1}{\sum }}\;a_{ii}-\frac{1}{n}\overset{n}{\underset{i=1}{\sum }}\;\overset{n}{\underset{\;j=1}{\sum }}\;a_{ij}$$

Therefore, the PSC in the special case is


(23) $$\rho =\frac{\mathrm{tr}(AM)}{\mathrm{tr}(M)}=\frac{1}{n-1}\left(\overset{n}{\underset{i=1}{\sum }}\;a_{ii}-\frac{1}{n}\overset{n}{\underset{i=1}{\sum }}\;\overset{n}{\underset{\;j=1}{\sum }}\;a_{ij}\right)$$

We do not need to re-estimate the genetic variance for the current population; rather, we can simply estimate the genetic variance that represents the base population with the typical REML method and then convert that variance into the genetic variance of the current population with PSC.

### Special family structures

**Monozygotic twins (MZT):** We first consider the genetic variance of a population consisting of *m* pairs of identical twins. We assume that parents are genetically unrelated both between families and within families. For one family, the genetic values of the twins are identical, although they are counted as 2. The within-family variance is zero. Genetic variance of the population is entirely contributed by the between-family variance. For *m* families, the total sample size is n=2m. Assume that the genetic variance of the base population (parents) is $\sigma_{A}^{2}$, the genetic variance of the current population is $\sigma_{MZT}^{2}=\rho_{MZT}\sigma_{A}^{2}$, where


(24) $$\rho_{MZT}=\frac{2(m-1)}{2m-1}$$

is PSC. When m=2, $\rho_{MZT}=2/3$. Suppose that one uses a statistical method, such as REML, to estimate the genetic variance correctly; that variance is not the genetic variance of the current population. The trajectory of $\rho_{MZT}$ over the number of families is shown in Fig. 1a. One can ignore the correction when m≥25 since $\rho_{MZT}\geq 0.98$. Because different families are assumed to be independent, the consequence of adding more families is the increase of PSC.

**Fig. 1.**
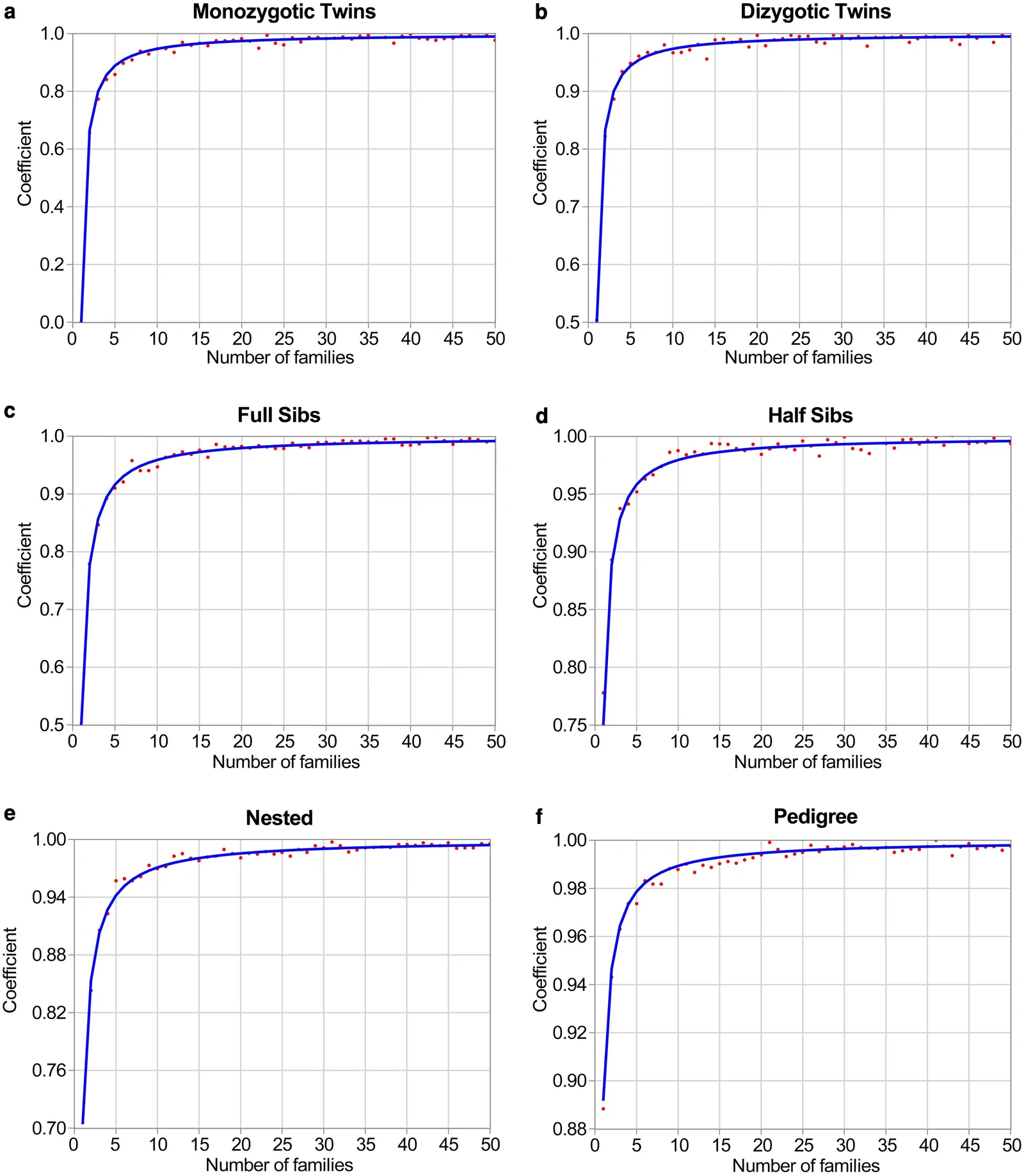
Pedigree sparsity coefficient (*ρ*, smooth curve) and simulated genetic variance (dot) against the number of families. a: MZT, b: DZT, c: Full sibs, d: Half sibs, e: Nested design, f: Pedigree

**Dizygotic twins (DZT):** Dizygotic twins are full sibs except that the family size is 2 (a constant). The PSC is


(25) $$\rho_{DZT}=\frac{4m-3}{2(2m-1)}$$

For one family, $\rho_{DZT}=0.5$, and the genetic variance of the current population is immediately cut to half. Figure 1b shows the trajectory of the change of $\rho_{DZT}$ over the number of families.

**Full sib families:** Let *k* be the number of full siblings per family and *m* be the number of families so that the total sample size is n=mk. The PSC of the current population consisting of *m* full-sib families is


(26) $$\rho_{FULL}=\frac{mk-[1+r_{FULL}(k-1)]}{mk-1}$$

where $r_{FULL}=0.5$ is the additive relationship between full sibs. Figure 1c shows the trajectory of the change of $\rho_{FULL}$ over the number of families when the family size is k=5. If the family size varies, say $k_{i}$ for the *i*th family, the PSC is


(27) $$\rho_{FULL}=\frac{1}{n-1}\left\{n-\frac{1}{n}\overset{m}{\underset{i=1}{\sum }}\;k_{i}[1+r_{FULL}(k_{i}-1)]\right\}$$

where $n=\overset{m}{\underset{i}{\sum }}\;k_{i}$ is the population size.

**Half sib families:** The PSC for half-sib families is


(28) $$\rho_{HALF}=\frac{mk-[1+r_{HALF}(k-1)]}{mk-1}$$

where $r_{HALF}=0.25$ is the additive relationship between half-sibs, *k* and *m* are the family size and number of families, respectively. Figure 1d shows the trajectory of the change of $\rho_{FULL}$ over the number of families when k=5. For variable family size, the PSC is


(29) $$\rho_{HALF}=\frac{1}{n-1}\left\{n-\frac{1}{n}\overset{m}{\underset{i=1}{\sum }}\;k_{i}[1+r_{HALF}(k_{i}-1)]\right\}$$

where $k_{i}$ is the size of the *i*th family and $n=\overset{m}{\underset{i}{\sum }}\;k_{i}$ is the population size.

**Full sib families nested within half sib families (the nested design):** Let *m* be the number of sire families, *d* be the number of dams per sire and *s* be the number of siblings per dam. The population size is n=mds. The PSC is


(30) $$\rho_{NEST}=\frac{1}{n-1}\{n-[1+r_{FULL}(s-1)+r_{HALF}s(d-1)]\}$$

Figure 1e shows the trajectory of change in $\rho_{NEST}$ over the number of sire families when d=5 and s=10. Let $d_{i}$ be the number of dams in the *i*th sire families, and $s_{ij}$ be the number of sibs for the *j*th dam within the *i*th sire. The population size is $n=\overset{m}{\underset{i=1}{\sum }}\;\overset{d_{i}}{\underset{\;j=1}{\sum }}\;s_{ij}$. The general formula for the unbalanced nested design is


(31) $$\rho_{NEST}=\frac{1}{n-1}\left\{n-\frac{1}{n}\overset{m}{\underset{i=1}{\sum }}\;\left[\overset{d_{i}}{\underset{\;j=1}{\sum }}\;s_{ij}+r_{FULL}\overset{d_{i}}{\underset{\;j=1}{\sum }}\;s_{ij}(s_{ij}-1)+r_{HALF}\overset{d_{i}-1}{\underset{\;j=1}{\sum }}\;\overset{d_{i}}{\underset{\;j^{\prime }=j+1}{\sum }}\;s_{ij}s_{ij^{\prime }}\right]\right\}$$

where $r_{FULL}=0.5$ and $r_{HALF}=0.25$.

**Pedigree data:** The additive relationship between individuals is complicated and there is no recognizable pattern that allows us to derive an explicit formula. Fortunately, we can use matrix algebra to derive the formula. Let *m* be the number of pedigrees in a population. Let $A_{i}$ be the additive relationship matrix of $k_{i}$ members within the *i*th pedigree for i=1,⋯,m. The PSC is


(32) $$\rho_{PED}=\frac{1}{n-1}\overset{m}{\underset{i=1}{\sum }}\;\left[\mathrm{tr}(A_{i})-\frac{1}{n}1^{T}A_{i}1\right]$$

where $n=\overset{m}{\underset{i=1}{\sum }}\;k_{i}$ is the population size, 1 is a unity vector of size $k_{i}$ and $1^{T}A_{i}1$ is the sum of all elements of matrix $A_{i}$. Figure 2 shows a pedigree with k=58 individuals. The additive relationship matrix of the 58 individuals is given in Supplementary Data 1. We now duplicate this pedigree *m* times to make a population and show the trajectory of the PSC (Fig. 1f). Since different pedigrees are assumed to be independent, adding more pedigrees to the population increases the pedigree sparsity coefficient.

**Fig. 2.**
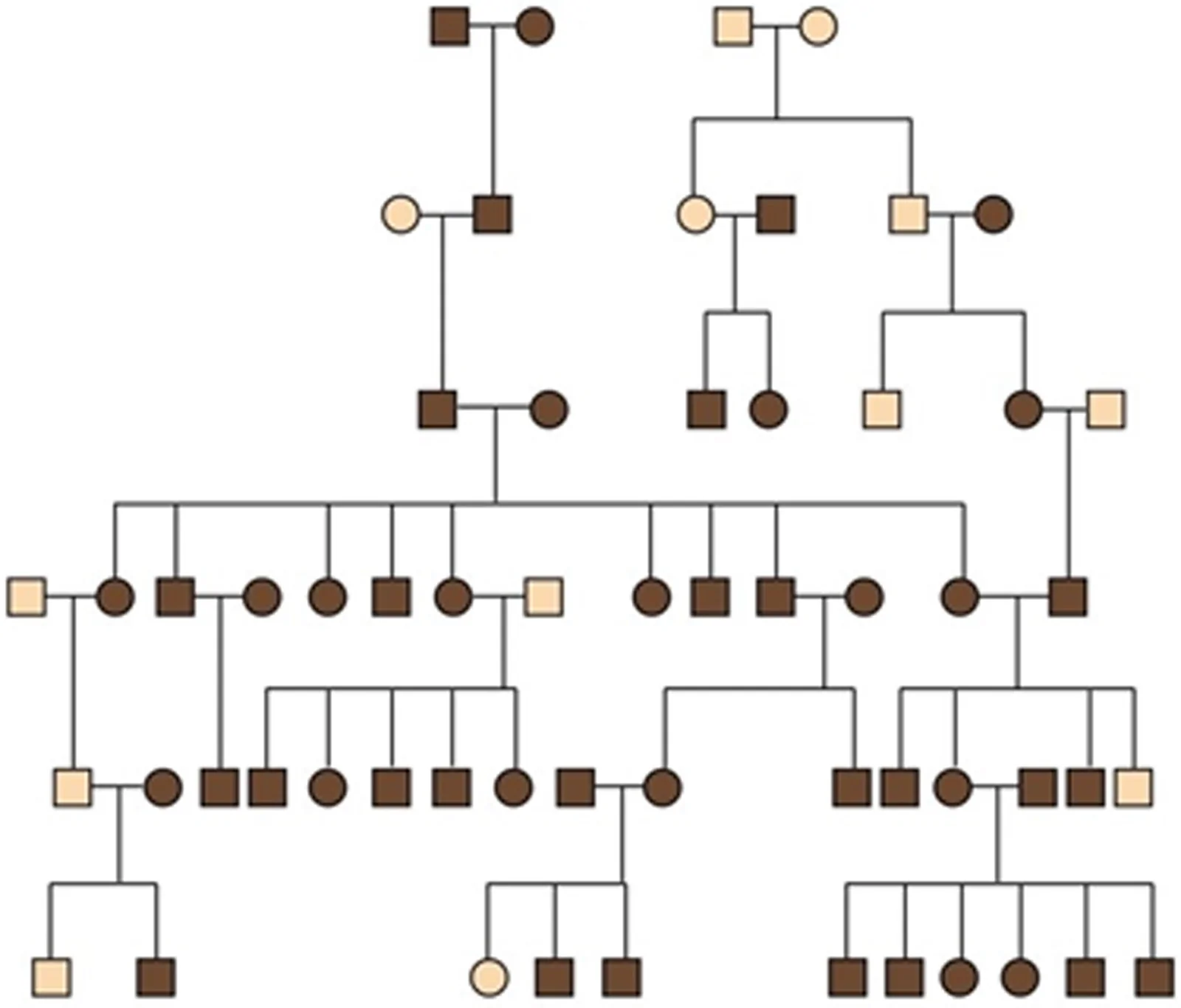
Pedigree used in the simulation study.

### REML estimate of genetic variance

The 2 variance components, $\theta =\{\sigma_{A}^{2},\sigma_{E}^{2}\}$, are estimated using the REML method with PROC MIXED or the mixed model function in R (Supplementary Code 1). The restricted log likelihood function is


(33) $$L_{R}(\theta )=-\frac{1}{2}\ln|V|-\frac{1}{2}\ln|X^{T}V^{-1}X|-\frac{1}{2}(y-X\hat{\beta }{)}^{T}V^{-1}(y-X\hat{\beta })$$

where


(34) $$\hat{\beta }=(X^{T}V^{-1}X{)}^{-1}X^{T}V^{-1}y$$

is the weighted least square estimates of *β*, and $\hat{\beta }$ is not a parameter but simply a function of parameter *θ* in the form of $V=A\sigma_{A}^{2}+I\sigma_{E}^{2}$.

### Genomic heritability

The pedigree and family data analysis for observed populations can be extended to genomic heritability. The linear mixed model is


(35) y=Xβ+g+ε

where $g∼N(0,G\sigma_{G}^{2})$ is the genomic values of all individuals in the sample, *G* is a genomic relationship matrix computed from genome-wide markers, $\sigma_{G}^{2}$ is the genomic variance, $\varepsilon ∼N(0,I\sigma_{E}^{2})$ is a vector of environmental effects and $\sigma_{E}^{2}$ is the environmental variance. Again, the genomic variance $\sigma_{G}^{2}$ is not the genetic variance in the current population. The variance of the current population is obtained from partitioning of the phenotypic variance,


(36) $$\sigma_{P}^{2}=\frac{1}{\mathrm{tr}(M)}y^{T}My=\frac{1}{\mathrm{tr}(M)}[\mathrm{tr}(GM)\sigma_{G}^{2}+\mathrm{tr}(M)\sigma_{E}^{2}]=\rho \sigma_{G}^{2}+\sigma_{E}^{2}$$

where ρ=tr(GM)/tr(M) is the genomic relationship sparsity coefficient and *M* is the fixed effect annihilator matrix, see equation (10). The genomic heritability in the current population is


(37) $$h_{G}^{2}=\frac{\rho \sigma_{G}^{2}}{\rho \sigma_{G}^{2}+\sigma_{E}^{2}}$$

### Validating the theoretical results via simulations

We performed Monte Carlo simulations to validate the theoretical derivations of PSC for different types of population. For each type of population, we set the genetic variance of the base population to $\sigma_{A}^{2}=1.0$. The genetic variance of the current population consisting of *m* families is


(38) $$\sigma_{X}^{2}(m)=\rho_{X}(m)\sigma_{A}^{2}$$

where X={MZT,DZT,FULL,HALF,NEST,PED} takes one of the 6 types of population and *m* is the number of independent families within the same type of population. The number of families varied starting from m=1 to m=50. The simulation was replicated 1000 times under each scenario. The average genetic variance over 1000 replicates was compared to the theoretical value calculated from equation (38). Since $\sigma_{A}^{2}=1.0$, the theoretical genetic variance is $\sigma_{X}^{2}(m)=\rho_{X}(m)$, which is a special case when the genetic variance of the base population is unity. The genetic values of the founders of each family were sampled from $N(0,\sigma_{A}^{2})$. The genetic value of each progeny was simulated from


(39) $$a=(a_{S}+a_{D})/2+a_{W}$$

where $a_{S}$ and $a_{D}$ are the genetic values of the sire and the dam, respectively. The last term, $a_{W}$, is the within-family segregation effect simulated from $N(0,\sigma_{W}^{2})$, where the within-family segregation variance is


(40) $$\sigma_{W}^{2}=\left(1-\frac{1}{2}f_{S}-\frac{1}{2}f_{D}\right)\sigma_{A}^{2}$$

with $f_{S}$ and $f_{D}$ being the inbreeding coefficients of the sire and dam, respectively. In our simulation study, all founders are assumed to be independent and noninbred. For the pedigree data, some nonfounder individuals may have nonzero inbreeding coefficients, and we have incorporated these in the simulation experiments. The genetic variance was calculated from the simulated genetic values, $a={\left[\begin{array}{cccc} a_{1} & a_{2} & \cdots & a_{n} \end{array}\right]}^{T}$, with


(41) $$\sigma_{A}^{2}=\mathrm{var}(a)=\frac{1}{n-1}\overset{n}{\underset{\;j=1}{\sum }}\;(a_{j}-\bar{a}{)}^{2}$$

The simulated genetic variances of the current populations for all 6 types of population are scattered plotted against the number of families along with their theoretical values.

### Replicated simulations under 3 scenarios

#### Nested design

The nested design is defined as full sibs nested within half sib families. The population consisted of m=5 sires, each mated with d=5 dams, and each dam had s=10 progenies. The population size was n=mds=250. The genetic variance and environmental variance were set at $\sigma_{A}^{2}=\sigma_{E}^{2}=10$ so that the true heritability is $h^{2}=10/(10+10)=0.5$. The simulation was replicated 1000 times. The genetic variance estimated with REML is denoted by $\sigma_{REML}^{2}$, which represents the genetic variance of the base population ($\sigma_{A}^{2}$). The genetic variance adjusted by the PSC is $\sigma_{ADJ}^{2}=\rho_{NEST}\sigma_{REML}^{2}$ where $\rho_{NEST}=0.9444$. The simulated genetic variance calculated from equation (41) is denoted by $\sigma_{SIMUL}^{2}$. The genetic variance calculated with the difference between the phenotypic variance and the estimated environmental variance is denoted by $\sigma_{DIFF}^{2}=\sigma_{P}^{2}-\sigma_{E}^{2}$.

#### Pedigree data

The pedigree presented in Fig. 2 was also used to simulate the data in the second scenario. The true genetic variance is $\sigma_{A}^{2}=10$ and the residual variance is $\sigma_{E}^{2}=10$ so that the true heritability is $h^{2}=\sigma_{A}^{2}/(\sigma_{A}^{2}+\sigma_{E}^{2})=0.5$. The simulation was replicated 1000 times. The genetic variance estimated with REML is $\sigma_{REML}^{2}$. The genetic variance of the current population is $\sigma_{ADJ}^{2}=\rho_{PED}\sigma_{REML}^{2}$, where


(42) $$\rho_{PED}=\frac{1}{n-1}\left[\mathrm{tr}(A)-\frac{1}{n}1^{T}A1\right]=0.8917$$

and *A* is the numerator relationship matrix of the pedigree (Supplementary Data 1).

#### Large population

The simulation was conducted with the simulatePed() function of the “pedSimulate.R” package (Nilforooshan 2022). The genetic relationship was calculated with the kinship() function of the “kisnhip2.R” package (Sinnwell et al. 2014). The large population consisted of n=1104 individuals generated from 30 discrete generations of random selection and random mating started with 500 independent founders. The current population of n=1104 were collected from the last 2 generations (562 individuals from generation 29 and 542 individuals from generation 30). The additive genetic relationship matrix of the 1104 individuals (matrix *A*) is given in Supplementary Data 2. To simulate the phenotypic values of the 1104 individuals, we generated the genetic effects (*a*) of the 1104 individuals from a multivariate normal distribution $a∼N(0,A\sigma_{A}^{2})$ where $\sigma_{A}^{2}=10$. We then simulated 1104 independent environmental effects from $\varepsilon ∼N(0,I\sigma_{E}^{2})$ where $\sigma_{E}^{2}=10$. The mean of the phenotypic values was μ=100 and no other fixed effects were simulated. The phenotypic values (*y*) were obtained from


y=1μ+a+ε

The annihilator matrix was


$$M=I-X(X^{T}X{)}^{-1}X^{T}$$

where X=1 (a unity vector of ones with length of 1104). The PSC of the population was


$$\rho =\frac{\mathrm{tr}(AM)}{\mathrm{tr}(M)}=\frac{\mathrm{tr}(AM)}{n-1}=\frac{\mathrm{tr}(AM)}{1103}=0.9859$$

With the same *A* matrix, the simulation was replicated 1000 times. The mixed() R function (Supplementary Code 1) was used to estimate the REML variance components. Since we kept the replicated *y* and *a*, we were able to calculate the genetic variance from the SIMUL method.

### Multiple generation simulation study

We simulated a multigeneration population starting with 100 founders (generation 0) to generation 50 with nonoverlapping generations. The simulation was conducted with the simulatePed() function of the “pedSimulate.R” package (Nilforooshan 2022). The genetic relationship was calculated with the kinship() function of the “kisnhip2.R” package (Sinnwell et al. 2014). Starting with 100 founders with 50 males and 50 females per generation, random mating pairs produced 2 progenies on average. Because of the random assignment of sex, the population size progressively decreased to 30 individuals in the last generation. The simulated pedigree in the tabular form is given in Supplementary Data 3. The true genetic variance of the simulated trait was $\sigma_{A}^{2}=10$ and the environmental variance was $\sigma_{E}^{2}=10$ so that the trait heritability in the base population was $h^{2}=0.5$. The simulation was replicated 1000 times and the average genetic variance per generation were obtained with 4 methods (REML, ADJ, DIFF and SIMUL).

### Long-term selection experiment of laboratory mice

Data from a long-term selection experiment in house mice were used to illustrate the application of the theory to estimating heritability (Careau et al. 2013). The experiment was divided into 8 lines (sub-populations), 4 of which were selected to increase voluntary wheel running and the remaining 4 lines served as controls (without selection). The total population size was n=17,988 with a generation average sample size of about 556. The trait was the wheel running (number of revolutions on day 5) after standardization (RUNs). The additive relationship matrix was computed with the kinship() function in the “kinship2.R” package (Sinnwell et al. 2014) for the entire population from generation −1 to generation 31, a very deep pedigree per line. Genetic and environmental variances of the wheel running trait were estimated for each generation but with the deep pedigree relationship matrix. As the pedigree became deeper, the generation-wise PSC ($\rho_{PED}$) became smaller (from 0.95 to 0.65). We now have 3 methods to calculate the genetic variance: $\sigma_{REML}^{2}$ is the estimated genetic variance with REML, $\sigma_{ADJ}^{2}=\rho_{PED}\sigma_{REML}^{2}$ is the adjusted genetic variance and $\sigma_{DIFF}^{2}=\sigma_{P}^{2}-\sigma_{E}^{2}$ is the difference between the observed phenotypic variance and the estimated residual variance.

Unlike the simulation studies where no fixed effects were involved other than the model intercept, in the mouse population, the fixed effects include the intercept ($X_{0}$), the sex effect ($X_{1}$) and the line effects ($X_{2}-X_{8}$) with the overall *X* matrix having *n* rows and 9 columns


(43) $$X=\left[\begin{array}{ccccccccc} X_{0} & X_{1} & X_{2} & X_{3} & X_{4} & X_{5} & X_{6} & X_{7} & X_{8} \end{array}\right]$$

where *n* is the generation-wise sample size that varies across generations. The generation specific PSC is $\rho_{PED}=\mathrm{tr}(AM)/\mathrm{tr}(M)$, where *A* is the generation-wise additive relationship matrix among individuals, $M=I-X(X^{T}X{)}^{-}X^{T}$ is the generation-wise annihilator matrix, and *X* is the generation-wise design matrix defined in equation (43) but only at that generation. The generation-wise genetic variance is the within-line genetic variance after we fit the fixed effects. The pedigree and the phenotypes are given in Supplementary Data 4.

### Genomic heritability

A rice population consisting of 210 recombinant inbred lines (RIL) was used to demonstrate genomic heritability estimates with 1619 SNP bins of the rice genome (Yu et al. 2011). Seven agronomic traits were analyzed, grain length (GL), grain number (GN), grain width (GW), heading date (HD), thousand-grain weight (KGW), tiller number per plant (TP), and grain yield (YD). The markers were used to construct the genomic relationship (IBS) matrix,


(44) $$G=\frac{1}{d}\overset{m}{\underset{k=1}{\sum }}\;Z_{k}Z_{k}^{T}$$

where $Z_{k}$ is an n×1 vector of binary indicators of the reference allele (0 for *aa* and 1 for *AA*) and *d* is a normalization factor to make the diagonal elements of matrix *G* as close to unity as possible. Note that there are only 2 alleles in RILs because all RILs are homozygotes. The REML method was used to estimate the polygenic variance and the residual variance for each trait. The marker inferred relationship matrix along with the 7 traits are presented in Supplementary Data 5. The heritability was obtained as follows,


(45) $$h_{REML}^{2}=\frac{\sigma_{REML}^{2}}{\sigma_{REML}^{2}+\sigma_{E}^{2}}$$

After adjustment for reduction from a base population to the current population, the heritability was


(46) $$h_{ADJ}^{2}=\frac{\sigma_{ADJ}^{2}}{\sigma_{ADJ}^{2}+\sigma_{E}^{2}}=\frac{\rho_{G}\sigma_{REML}^{2}}{\rho_{G}\sigma_{REML}^{2}+\sigma_{E}^{2}}$$

where


(47) $$\rho_{G}=\frac{1}{n-1}\left(n-\frac{1}{n}1^{T}G1\right)=\frac{1}{210-1}(210-110.2683)=0.477185$$

Heritability obtained from the difference method is


(48) $$h_{DIFF}^{2}=\frac{\sigma_{DIFF}^{2}}{\sigma_{DIFF}^{2}+\sigma_{E}^{2}}$$

where $\sigma_{DIFF}^{2}=\sigma_{P}^{2}-\sigma_{E}^{2}$. The adjusted polygenic variance is less than half of the REML estimated polygenic variance. The phenotypic variance is a fixed effect adjusted variance, $\sigma_{P}^{2}=y^{T}My/\mathrm{tr}(M)$.

## Results

### Validation of genetic variances for various types of population

The average genetic variance from 1000 replicated simulations was compared with the theoretical value for each type of population from one family to 50 families (Fig. 1). The trajectories of the simulated genetic variances (dots) aligned with theoretical values (curves) very nicely. This validated both the theory and the simulation program. The *y*-axis of Fig. 1 is the estimated genetic variance $\sigma_{X}^{2}=\rho_{X}\sigma_{A}^{2}=\rho_{X}$, where $\sigma_{A}^{2}=1$ and thus the axis was labeled “Coefficient”. Both MZT and DZT have the same family size (k=2), but the PSC reached 1 at different speeds with the DZT being sparser than the MZT and thus reaching 1 faster than the MZT. For example, when the number of families is 5, $\rho_{DZT}\approx 0.95$, but $\rho_{MZT}\approx 0.90$ (Fig. 1, a and b). The same trend was also observed for the sib families, with the half-sib family reaching 1 faster than the full-sib family because the PSC of the latter is higher than the former (Fig. 1, c and d). The nested family structure and the pedigree data show similar trends (Fig. 1, e and f). In general, the PSC reaches 0.98 with about 30 families in all types of populations.

### Replicated simulations under 3 scenarios

#### Nested design

The average parameters from 1000 replicated simulations are illustrated in Fig. 3 for the nested design, with Fig. 3a for heritability, Fig. 3d for genetic variance, and Fig. 3g for the true values of genetic variance. The reduction of the parameters from REML to the 3 methods (ADJ, DIFF, SIMUL) is obvious. The true parameter values (Fig. 3g) are $\sigma_{A}^{2}=10$ for the REML method and


$$\sigma_{ADJ}^{2}=\sigma_{DIFF}^{2}=\sigma_{SIMUL}^{2}=\rho \sigma_{A}^{2}=0.9444\times 10=9.444$$

for the other methods. Supplementary Note 1 describes the distributions of the genetic parameters from 1000 replicated simulations with both the histograms (Supplementary Fig. 1, a and b) and the boxplots (Supplementary Fig. 1, c and d). The estimated genetic variances and heritability for the 4 methods from the 1000 replicated samples are given in Supplementary Data 6 for interested readers to investigate.

**Fig. 3.**
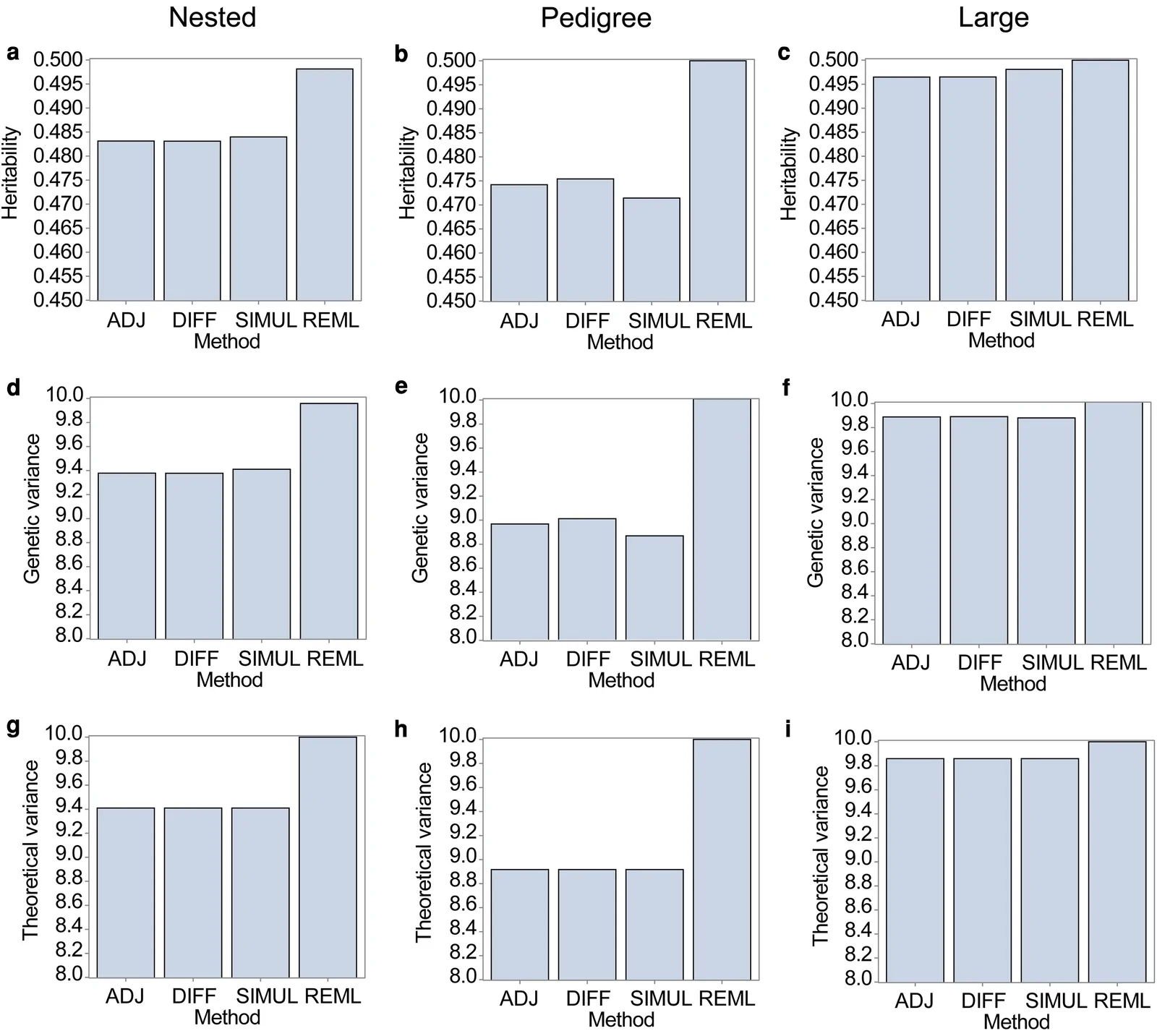
Comparison of REML estimate of heritability (a and b), genetic variance (c and d) and true genetic variance (e and f) for nested design (column 1), pedigree data (column 2) and the large population (column 3) from replicated simulation experiments.

#### Pedigree data

Figure 3 shows the average parameters from 1000 replicated simulations for the pedigree data (Fig. 3, b, e and h). The REML estimate is close to the true value ($\sigma_{A}^{2}=10$) while the other 3 methods produced genetic variances around the expected value


$$\sigma_{X}^{2}=\rho_{X}\sigma_{A}^{2}=0.8917\times 10=8.917$$

Among the 3 methods, the SIMUL method is the most accurate one, closer to the true value. In reality, this method does not exist and is only available for simulation studies. Compared with the SIMUL method, the ADJ and DIFF methods slightly overestimated the genetic variance and the heritability (Fig. 3, b and e). Supplementary Note 1 describes the distributions of the genetic parameters from 1000 replicated simulations with both the histograms (Supplementary Fig. 2, a and b) and the boxplots (Supplementary Fig. 2, c and d). The estimated genetic variances and heritability for the 4 methods from the 1000 replicated simulations are given in Supplementary Data 7 for interested readers to investigate.

#### Large population

Figure 3 shows the average parameters from 1000 replicated simulations for the large population (Fig. 3, c, f and i). The REML estimate is close to the true value ($\sigma_{A}^{2}=10$) while the other 3 methods produced genetic variances around the expected value


$$\sigma_{X}^{2}=\rho_{X}\sigma_{A}^{2}=0.9859\times 10=9.859$$

Compared with the SIMUL method, the ADJ and DIFF methods slightly overestimated the heritability (Fig. 3c). Supplementary Note 1 describes the distributions of the genetic parameters from 1000 replicated simulations with both the histograms (Supplementary Fig. 3, a and b) and the boxplots (Supplementary Fig. 3, c and d). The estimated genetic variances and heritability for the 4 methods from the 1000 replicated simulations are given in Supplementary Data 8 for interested readers to investigate.

### Multiple generation simulations

The generation-wise population size is plotted in Fig. 4a, showing that the population size started at 100 for generation 0 and decreased to about 30 at generation 50. The PSC is illustrated in Fig. 4b, starting from $\rho_{PED}=1.0$ (generation 0) to $\rho_{PED}=0.75$ (generation 50). The genetic variances per generation are illustrated in Fig. 4c, where $\sigma_{REML}^{2}$ is the estimated genetic variance with the REML method, which varies around the true value of $\sigma_{A}^{2}=10$. There is a trend of slightly greater than 10 toward the end of the simulation. This deviation is due to the small sample size after generation 35. Although we replicated the experiment 1000 times, the pedigree was only simulated once. The other 3 methods ($\sigma_{ADJ}^{2}$, $\sigma_{DIFF}^{2}$ and $\sigma_{SIMUL}^{2}$) are all decreased progressively as the pedigree becomes denser and denser. The red curve ($\sigma_{SIMUL}^{2}$) represents the “true genetic variance” because we directly calculated the variance of the simulated genetic values as shown in equation (41). The blue and green curves represent the adjusted genetic variance ($\sigma_{ADJ}^{2}$) and the difference between phenotypic variance and the environmental variance ($\sigma_{DIFF}^{2}$), both of which were obtained from the pedigree and the phenotype data. The conclusion from this simulation is that the PSC for genetic variance is very effective and reliable. As the number of generations progressively increases, the pedigree density coefficient increases and thus the observed genetic variance decreases. This is opposite to the situation where the pedigree sparsity increases as the number of independent families increases.

**Fig. 4.**
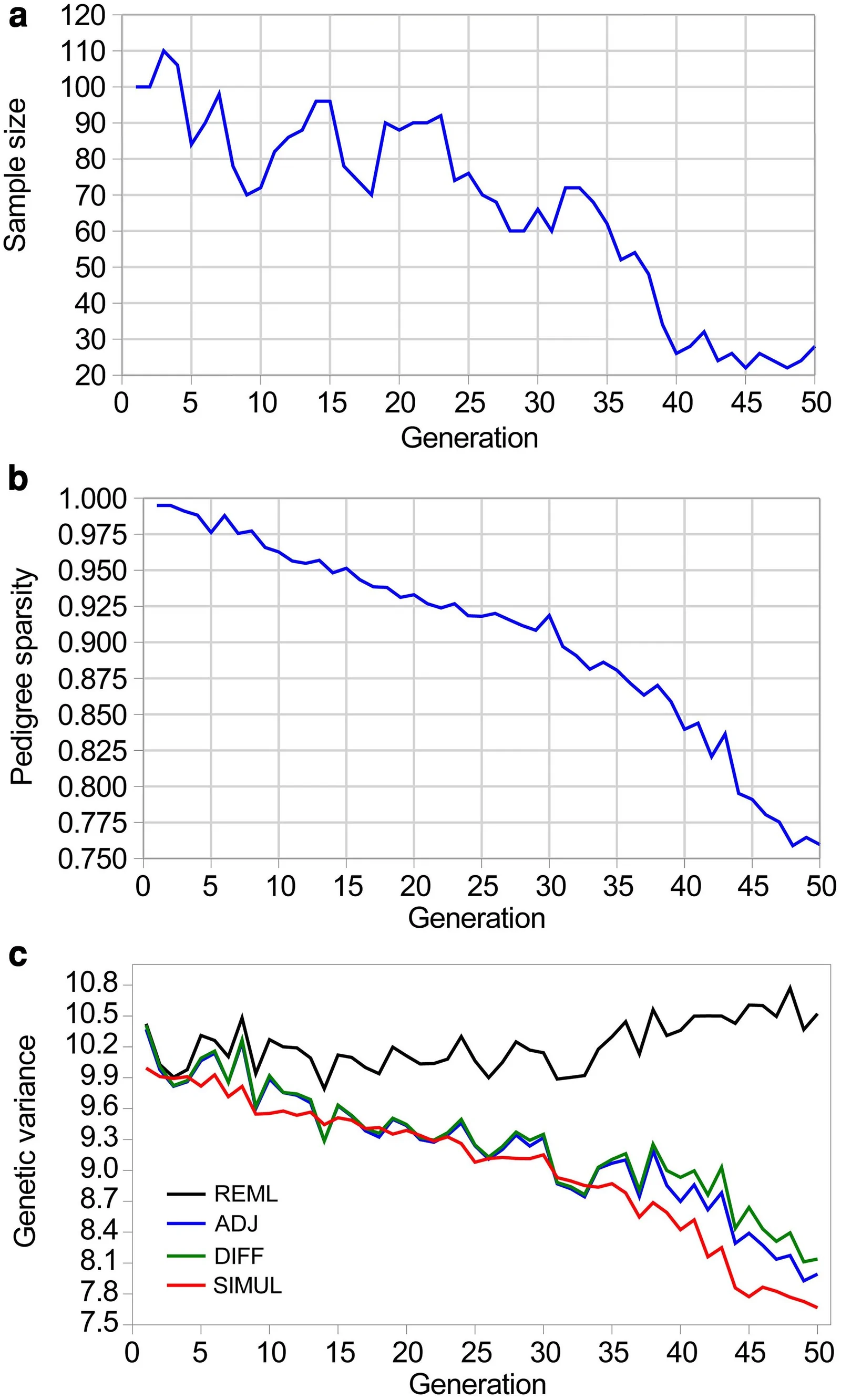
Multiple generation simulation study. a) sample size per generation; b) pedigree sparsity coefficient; c) genetic variance.

### Long-term selection experiment of laboratory mice

Figure 5a shows the population size across all 32 generations with a generation-wise population size of about 556. Figure 5b shows that as the pedigree became deeper, the PSC ($\rho_{PED}$) became smaller (from 0.95 to 0.65). We used 3 methods to calculate the genetic variance: $\sigma_{REML}^{2}$ is the estimated genetic variance with REML, $\sigma_{ADJ}^{2}=\rho_{PED}\sigma_{REML}^{2}$ is the adjusted genetic variance and $\sigma_{DIFF}^{2}=\sigma_{P}^{2}-\sigma_{E}^{2}$ is the difference between the observed phenotypic variance and the estimated residual variance. Figure 5c illustrates the estimated genetic variance ($\sigma_{REML}^{2}$), the variance after adjustment ($\sigma_{ADJ}^{2}$) and the variance taking the difference between the phenotypic variance and the environmental variance ($\sigma_{DIFF}^{2}$). The apparent cyclical variation in genetic variance is probably attributable to seasonal effects (Careau et al. 2013). The REML-estimated genetic variance is consistently higher than the adjusted variance and the variance of difference. The $\sigma_{ADJ}^{2}$ and $\sigma_{DIFF}^{2}$ are remarkably consistent, further validating the theory.

**Fig. 5.**
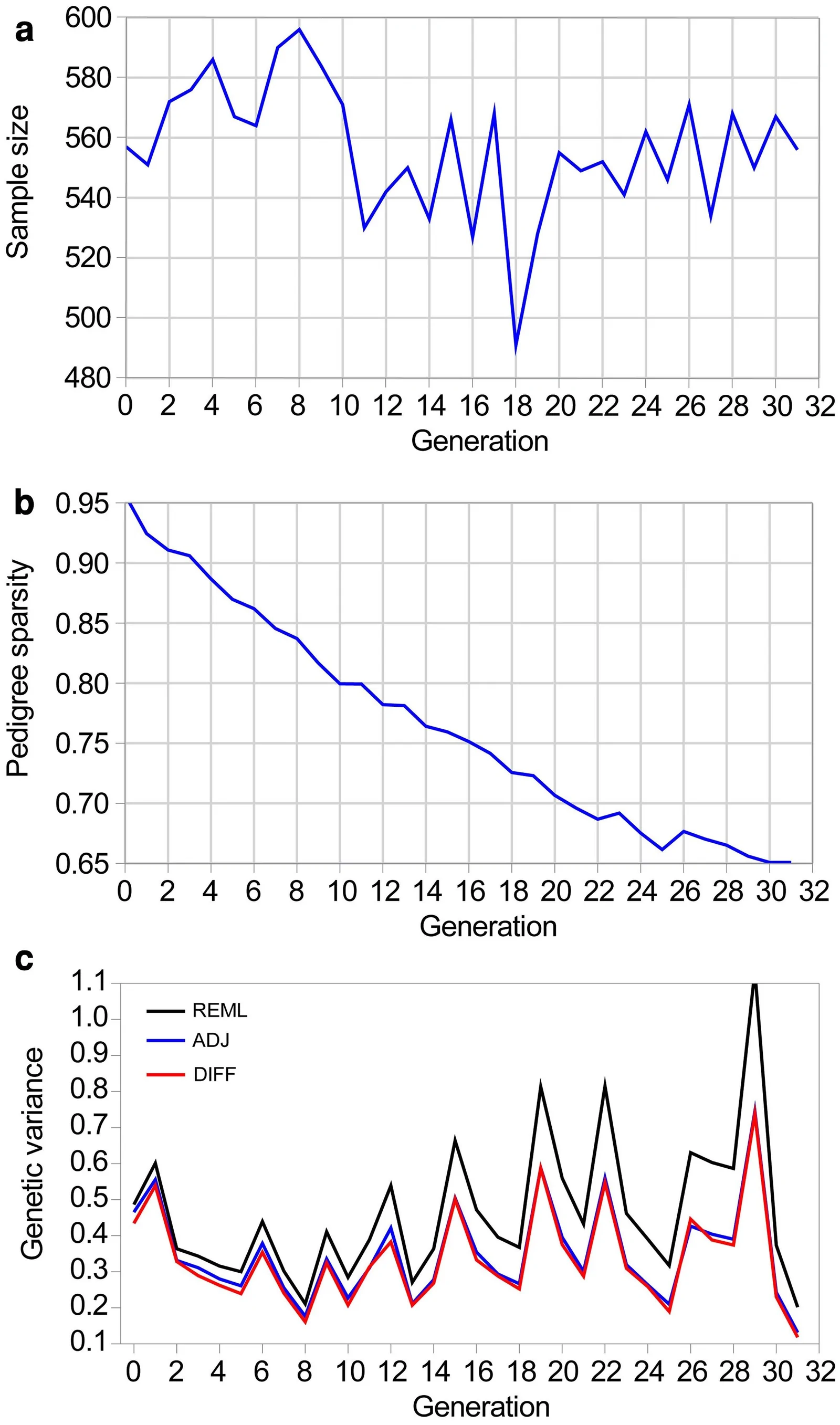
Multiple generation selection experiment in house mice. a) sample size per generation; b) pedigree sparsity coefficient; c) genetic variance.

### Genomic heritability of a rice population

From the genomic relationship matrix (*G*), we calculated the PSC, which is ρ=0.477185. Therefore, the estimated polygenic variance from the REML method was cut to half to represent the genetic variance in the current population. Figure 6 compares the genetic variances from the 3 methods for all 7 traits. Again, $\sigma_{ADJ}^{2}$ is remarkably close to $\sigma_{DIFF}^{2}$. Genomic heritability defined via the linear mixed model with marker inferred relationship matrix does not represent the heritability of the current population. Adjustment is required to convert the heritability into genomic heritability of the current population.

**Fig. 6.**
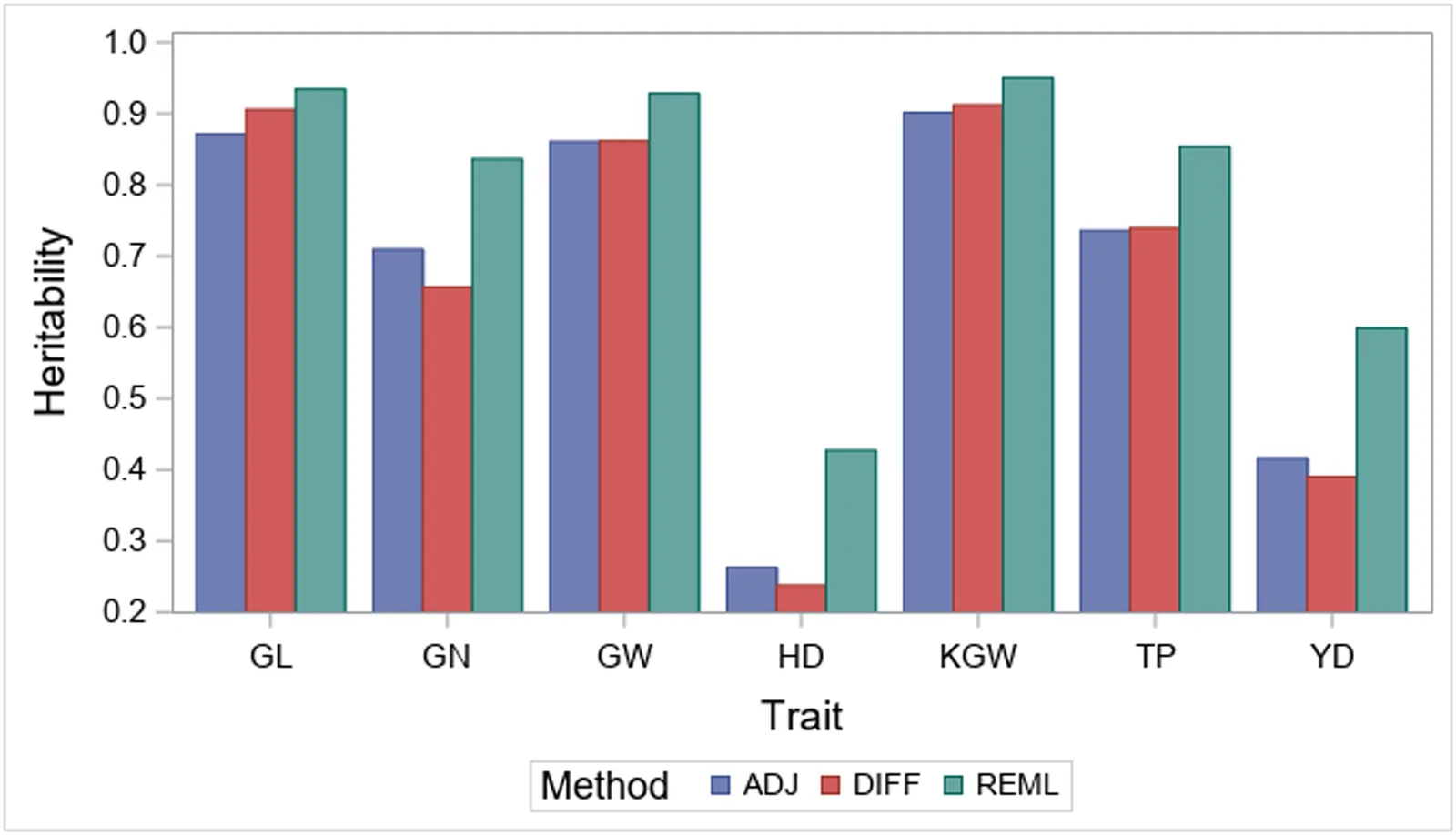
Genomic heritability for 7 traits of a rice population.

### Large human populations

Some human populations do not have very deep pedigrees, e.g. studying trios, which is one of the foundational designs in human genetics. A trio refers specifically to a family unit consisting of 2 biological parents and one child. Suppose that a population consists of 1000 trios, making a total population size of 3000. The additive relationship matrix of a trio is


$$A=\left[\begin{array}{ccc} 1 & 0 & 0.5\\ 0 & 1 & 0.5\\ 0.5 & 0.5 & 1 \end{array}\right]$$

where the child is listed as the last individual of the trio. From equation (32), the PSC of such a population is


(49) $$\rho_{PED}=\frac{1000}{3000-1}\left[\mathrm{tr}(A)-\frac{1}{3000}1^{T}A1\right]=\frac{1000}{3000-1}\left[3-\frac{1}{3000}(3+0.5\times 4)\right]=0.9997777$$

Such a high PSC makes the adjustment of estimated genetic variance unnecessary.

## Discussion

Heritability can also be estimated with the parent–offspring regression analysis, where the phenotypic value of the offspring is treated as the response variable and the phenotypic value of the parent treated as the independent variable. If the parents are not related, the estimated heritability is the heritability of the offspring population, which is current. However, if parents are included and counted together with the offsprings as the current population, the heritability is no longer the heritability of the current population. The PSC is the same as that of dizygotic twins.

Heritability estimated with deep pedigrees represents the heritability in a base population. This study shows that heritability estimated from family data (shallow pedigrees) is not the variance of the current population either. To better represent the heritability of the current population, Careau et al. (2013) suggested treating the parents as independent and recalculating the additive relationship matrix of the current population. Although this heritability is not the heritability of the base population, it is not the heritability of the current population either. With the PSC adjustment developed here, we simply estimate the genetic variance using the deep pedigrees and convert the genetic variance into the one representing the current population. A Bayesian method has been used to estimate generation-wise genetic variance (Sorensen et al. 2001).

The PSC or PDC is sufficiently general and applies to any complicated pedigrees. The systematic family structures are only special cases of pedigrees; it is called the animal model (Henderson 1976). The REML estimated genetic variance is for a base population, but the estimated environmental variance is for the current population. Therefore, the difference between the fixed effect adjusted phenotypic variance and the environmental variance provides a validation for the application of PSC. We investigated the simplest situation where only additive genetic variance, and thus the narrow sense heritability, are considered. The model can be easily extended to dominance and epistatic model, which is described in Supplementary Note 2.

The original definition of genetic variance applies to the current population because the genetic effects are treated as fixed effects and the genotype array reflect the variation among individuals within the population, but the estimated genetic variance is for a base population. Such a genetic paradox exists for more than a century since the concept of genetic variance proposed by Fisher (Fisher 1918), which was called the narrow sense heritability (Lush 1940). The genetic paradox is now coming to an end. The paradox does not exist for plant breeding where genotypes can be replicated either in the form of homozygotes or immortalized heterozygotes. It happens in humans and animals because the genotypic effect and environmental effect within an individual cannot be separated. The monozygotic twin's data, if analyzed properly, may avoid the paradox. Just like plants, twins are treated as one genotype replicated twice. We can estimate the environmental variance as the within-family variance and the between-family variance is the genetic variance. This genetic variance is the genetic variance of the current population when all individuals are independent. In animals, if a trait can be repeatedly measured, eg milk yield in dairy cows and the bristle numbers of 2 sides of Drosophila, the estimated repeatability is considered as the upper limit of the heritability. This heritability is the heritability of the current population if all individuals are independent.

The pedigree sparsity coefficient may be considered as a tool to convert an n×n dense pedigree to an m×m sparse pedigree (identity matrix). This function is similar to calculating the effective population size. For example, a population consisting of *m* full-sib families will have an effective population size of


$$N_{e}=\frac{4N_{m}N_{f}}{N_{m}+N_{f}}=\frac{4mm}{m+m}=2m$$

where $N_{m}=N_{f}=m$. The effective population size depends only on the number of parents, not the number of progenies. However, the PSC depends on the number of families and the family sizes. Therefore, it is not obvious how to utilize *ρ* to calculate the effective population size. It is an interesting topic, but further studies are needed. Although *ρ* cannot be used to estimate the effective population size, it can be used to estimate the effective number of relatives. Suppose that the observed number of individuals in a pedigree is *n*, the effective number of relatives is


$$n_{e}=\rho n=\frac{\mathrm{tr}(AM)}{\mathrm{tr}(M)}\;n$$

For example, the rice population presented earlier has n=210 individuals, but the effective number of relatives is $n_{e}=0.477185\times 210\approx 100.212$. The simulated large human population presented in the result section has a population size of n=1104. The effective number of relatives is $n_{e}=0.985959\times 1104\approx 1088$.

The numerator relationship matrix calculated from pedigree data starts from a founder (base) population where individuals are assumed to be unrelated and noninbred. Therefore, the base population is arbitrarily defined, and it is a relative term. Suppose that a base population contains inbred individuals who are also genetically related; if the founder relatedness has been incorporated into the algorithm when the numerator relationship matrix is calculated, the pedigree is denser than a pedigree when the founders are independent. The estimated genetic variance from such a dense relationship matrix represents the genetic variance of another hypothetical “base population” where individuals are independent. The usual practice is to ignore the relatedness of the founders when the numerator relationship matrix is calculated. The estimated genetic variance from such a relationship matrix is biased downward because of the ignorance of related founders. In a long-term selection experiment, the pedigree within a line starts at the base population and relatedness of individuals cumulates generation by generation. Genetic variance can be estimated from individuals of each generation, but the relationship matrix of the individuals within the generation is calculated cumulatively. The generation-wise genetic variance reflects the genetic variance of the base population, as demonstrated in the long-term selection experiment of mice (Careau et al. 2013). One potential consequence of related founders is that the pedigree of the current population may be sparser than the founder population so that PSC may be over the maximum value of 1. With the tabular algorithm for calculating the kinship matrix perhaps, this will rarely happen or may not happen at all.

Finally, although most pedigrees in human and farm animals may be sparse enough that the adjustment may not be much useful in practice, understanding the theoretical discrepancy is important, and it is the job of theoretical quantitative geneticists. The hybrid rice population study shows that the PSC is 0.4772, indicating that PSC may be important in estimating genomic heritability where the genomic relationship matrix is used.

## Supplementary Material

iyag232_Supplementary_Data

## Data Availability

The author affirms that all data necessary for confirming the conclusions of the article are present within the article, figures, and tables. Supplemental material is available at GENETICS online.
