# Supplementary material for: Heritability—a paradox of quantitative genetics
Source: Genetics. 2026 Aug 31;234(3):iyag232. doi: 10.1093/genetics/iyag232 (PMC13732719; doi:10.1093/genetics/iyag232)
Supplement: Supplementary file 1 [file iyag232_supplementary_data.zip › Note_S1_GENETICS-2026-309792.docx]

**Supplementary Note S1: Distributions of Estimated Genetic Parameters in Replicated Simulations**

**1 Full-sib families nested within half-sib families**

For the simulation experiment under the full-sib families nested with half-sib family’s structure, we presented the histograms and box plots of the 1000 replicates for the four methods (REML, ADJ, DIFF, SIMUL) in **Supplementary Fig S1**. The distributions of the estimated genetic variance (**Fig S1a**) show that three methods (ADJ, DIFF, REML) have skewed distribution towards the left while the distribution of the SIMUL method is more symmetrical with a very small standard deviation. This small standard deviation is because the true genetic value from each simulated individual was used to draw the plots. The distributions of the heritability are shown on **Fig S1b**, where all methods are more normal (symmetric). Again, the standard deviation of the SIMUL method is very small compared with the other methods. The box plots are shown on **Fig S1c** for the genetic variance and **Fig S1d** for the heritability. The results agree with the histograms, where the SIMUL method consistently shows much smaller interquartile range (IQR). The REML method consistently shows a higher mean than the other three methods. The estimated genetic variances and heritability of the four methods from the 1000 replicated simulations are given in **Supplementary Data S6** for interested readers to verify the results.

**2 Replicated pedigree data**

For the simulation experiment of the pedigree data, the histograms and box plots of the 1000 replicates for the four methods (REML, ADJ, DIFF, SIMUL) are shown in **Supplementary Fig S2**. The distributions of the estimated genetic variance (**Fig S2a**) show that the three methods (ADJ, DIFF, REML) have skewed distributions towards the left while the distribution of the SIMUL method is more symmetrical with a very small standard deviation. The distributions of the heritability are shown on **Fig S2b**. The box plots are shown on **Fig S2c** for the genetic variance and **Fig S2d** for the heritability. The results agree with the histograms, where the SIMUL method consistently shows much smaller interquartile range (IQR). The estimated genetic variances and heritability of the four methods from the 1000 replicated simulations are given in **Supplementary Data S7** for interested readers to verify the results.

**3 Replicated large population**

For the simulation experiment of the large population consisting of *n* = 1104 individuals, the histograms and box plots of the 1000 replicates for the four methods (REML, ADJ, DIFF, SIMUL) are shown in **Supplementary Fig S3**. The distributions of the estimated genetic variance (**Fig S3a**) show that all four methods (ADJ, DIFF, REML, SIMUL) have roughly normal distributions. The variation of the SIMUL method is much smaller than the other methods. The distributions of the heritability are shown on **Fig S3b**. The box plots are shown on **Fig S3c** for the genetic variance and **Fig S3d** for the heritability. The results agree with the histograms, where the SIMUL method consistently shows much smaller interquartile range (IQR). The estimated genetic variances and heritability of the four methods from the 1000 replicated simulations are given in **Supplementary Data S8** for interested readers to verify the results.
