# Supplementary material for: Heritability—a paradox of quantitative genetics
Source: Genetics. 2026 Aug 31;234(3):iyag232. doi: 10.1093/genetics/iyag232 (PMC13732719; doi:10.1093/genetics/iyag232)
Supplement: Supplementary file 1 [file iyag232_supplementary_data.zip › Note_S2_GENETICS-2026-309792.docx]

**Supplementary Note S2: Non-additive Model**

The model may be extended to cover dominance and epistatic variances with ease. Take the dominance variance as example, let the linear mixed model be

$$y=X\beta+Z\gamma+W\delta+\varepsilon$$

where $W$ is a design matrix for the dominance effects. Define $D=WW^{T}$ as the dominance relationship matrix, just like $A=ZZ^{T}$ defined earlier for the additive model. Let $\delta\sim N(0,D\sigma_{D}^{2})$and $\sigma_{D}^{2}$ be the dominance variance. We partition the total sum of squares into

$$E(y^{T}My)=E(\gamma^{T}Z^{T}MZ\gamma)+E(\delta^{T}W^{T}MW\delta)+E(\varepsilon^{T}M\varepsilon)$$

where

$$E(\gamma^{T}Z^{T}MZ\gamma)=tr(ZZ^{T}M)\sigma_{A}^{2}=tr(AM)\sigma_{A}^{2}$$

and

$$E(\delta^{T}W^{T}MW\delta)=tr(WW^{T}M)\sigma_{D}^{2}=tr(DM)\sigma_{D}^{2}$$

We also have

$$E(\varepsilon^{T}M\varepsilon)=tr(M)\sigma_{E}^{2}$$

Therefore, the total phenotypic variance, $\sigma_{p}^{2}=E(y^{T}My)/tr(M)$, is partitioned as

$$\sigma_{P}^{2}= \frac{1}{tr(M)}\left[tr(AM)\sigma_{A}^{2}+tr(DM)\sigma_{D}^{2}+tr(M)\sigma_{E}^{2}\right]= \rho_{A}\sigma_{A}^{2}+\rho_{D}\sigma_{D}^{2}+\sigma_{E}^{2}$$

where $\rho_{A}=tr(AM)/tr(M)$ and $\rho_{D}=tr(DM)/tr(M)$ are pedigree sparsity coefficients.

An epistatic model may contain all three epistatic effects, in addition to the additive and dominance effects. Let us rewrite the additive and dominance relationship matrices by $K_{A}=A$ and $K_{D}=D$. Let $X\circ Y$ be the Hadamard matrix product between matrix *X* and matrix *Y* (element-wise matrix multiplication). We can define the epistatic relationship matrices by $K_{AA}=A\circ A$, $K_{AD}=A\circ D$, and $K_{DD}=D\circ D$ for the additive by additive, additive by dominance and dominance by dominance matrices. The total phenotypic variance in the current population can be partitioned as

$$\sigma_{P}^{2}= \rho_{A}\sigma_{A}^{2}+\rho_{D}\sigma_{D}^{2}+\rho_{AA}\sigma_{AA}^{2}+\rho_{AD}\sigma_{AD}^{2}+\rho_{DD}\sigma_{DD}^{2}+\sigma_{E}^{2}$$

where $\rho_{AA}=tr(K_{AA}M)/tr(M)$ and so on. The additive-by-additive variance in the current population is $\rho_{AA}\sigma_{AA}^{2}$.
